# Advances in Xanthan Gum-Based Systems for the Delivery of Therapeutic Agents

**DOI:** 10.3390/pharmaceutics15020402

**Published:** 2023-01-25

**Authors:** Mahima Jadav, Deep Pooja, David J. Adams, Hitesh Kulhari

**Affiliations:** 1 School of Nano Sciences, Central University of Gujarat, Gandhinagar 382030, Gujarat, India; 2 School of Pharmacy, National Forensic Science University, Gandhinagar 382007, Gujarat, India; 3 Illawarra Health and Medical Research Institute (IHMRI), University of Wollongong, Wollongong, NSW 2522, Australia

**Keywords:** biopolymers, polysaccharides, drug delivery, controlled release, nanoparticles, physiochemical properties

## Abstract

In the last three decades, polymers have contributed significantly to the improvement of drug delivery technologies by enabling the controlled and sustained release of therapeutic agents, versatility in designing different delivery systems, and feasibility of encapsulation of both hydrophobic and hydrophilic molecules. Both natural and synthetic polymers have been explored for the delivery of various therapeutic agents. However, due to the disadvantages of synthetic polymers, such as lack of intrinsic biocompatibility and bioactivity, hydrophobicity, and expensive and complex procedure of synthesis, there is a move toward the use of naturally occurring polymers. The biopolymers are generally derived from either plants or microorganisms and have shown a wide range of applications in drug administration due to their hydrophilic nature, biodegradability, biocompatibility, no or low toxicity, abundance, and readily available, ease of chemical modification, etc. This review describes the applications of a biopolymer, xanthan gum (XG), in the delivery of various therapeutic agents such as drugs, genetic materials, proteins, and peptides. XG is a high molecular weight, microbial heteropolysaccharide and is produced as a fermented product of Gram-negative bacteria, *Xanthomonas campestris*. Traditionally, it has been used as a thickener in liquid formulations and an emulsion stabiliser. XG has several favourable properties for designing various forms of drug delivery systems. Furthermore, the structure of XG can be easily modified using different temperature and pH conditions. Therefore, XG and its derivatives have been explored for various applications in the food, pharmaceutical, and cosmetic industries.

## 1. Introduction

Natural polymers and their derivatives possess properties such as biocompatibility, biodegradability, high availability, and capability for chemical modifications, which makes them ideal for new drug delivery systems. Natural polymers are scientifically chosen over synthetic polymers due to their predictable outcomes, such as biodegradability and biocompatibility in the human body. Natural gums are made up of several sugar units connected by the glycosidic bonds known as polysaccharides. Plants comprising vascular tissues produce gums as a defence mechanism against any injury. The gums produced are heteropolysaccharides, but upon hydrolysis, they are converted into monosaccharides such as mannose, xylose, arabinose, glucose, uronic acid, and galactose [1]. These natural gums are extensively researched because they are biodegradable, biocompatible, and sustainable and are often used in different industries [2]. Natural gums, xanthan gum, locust bean gum, guar gum, and tara gum, have a comprehensive application in the pharmaceutical industries [3,4].

Xanthan gum (XG) is a naturally occurring extracellular heteropolysaccharide and is a fermentation product of *Xanthomonas campestris*, which is an aerobic, Gram-negative pathogen. Generally, the pathogen invades and multiplies in the plant vascular tissues of the crucifers such as cabbage, broccoli, and radish and produces V-shaped necrotic lesions at the foliar areas and darkens the veins, causing typical “black rot” symptoms. The bacterium invades the crucifers through stomata, roots, wounds, and hydathodes [5]. It can live in the soil for a year and spread through irrigation and surface water. The bacterium depends on the water for its survival [6]. The world has faced periodic epidemics of black rot disease, especially in agriculturally developing areas of Asia and Africa. Such incidences cause significant losses in agricultural products [5]. The average molecular weight of XG chains ranges from 1 × 10^6^ to 20 × 10^6^ g/mol, which varies according to its biosynthesis conditions and interchain linkages. It is a water-soluble carbohydrate with a cellulosic backbone and a side chain of β-D-mannose-β-D glucuronic acid-α-D-mannose [7].

XG is one of the most important commercially available microbial polysaccharides. In the 1950s, Allene Rosalind Jeanes, from the United States Department of Agriculture, discovered XG. After being approved by the United States Food and Drug Administration (FDA) in 1969 as a food additive (Fed. Reg. 345376), XG was extensively used in food industries as a thickener and a stabiliser. In the 1960s, Kelco company (CP Kelco, Atlanta, GA, USA) started the production of XG in the USA under the name Kelzan1. Jungbunzlauer Austria AG and Solvay in Europe, with the trade name of Rhodopol^®^, started the production of XG at an industrial scale. Since 2005, one of the biggest producers of XG has been China [8].

Due to its pseudoplastic behaviour and thermal stability, it is added to the water-based drilling fluids in the petroleum industry [9]. Essentially, XG plays an important role as an efficient thickener and favours emulsion stability in the food and cosmetic industries [10]. In the pharmaceutical industry, XG is considered an active excipient for the sustained release of drugs. Furthermore, being highly stable in a broad range of temperatures, pH, and ionic strength and having biocompatibility with the human body, it is used as a drug delivery system. XG is also used as a reducing and capping agent for nanoparticles to make them biodegradable and non-toxic [11].

This review offers a systematic and comprehensive summary of current advancements in XG modification for its use as an excipient in pharmaceutical formulation development. Further, the applicability of XG in the delivery of various therapeutic agents such as drugs, genetic materials, proteins, and peptides is highlighted.

## 2. Source and Physicochemical Properties of XG

Xanthan gum is an exopolysaccharide, mainly produced by a plant pathogenic Gram-negative bacterial genus *Xanthomonas*. *Xanthomonas* cells are usually found in a single rod shape with a single polar flagellum. Various strains of *Xanthomonas* are capable of production of XG, such as *X. arboricola, X. axonopodis, X. citri, X. phaseoli, X. vasculorium, X. gummisudans, X. axonopodis, X. fragaria, X. campestris*. Amongst all the species, *X. campestris* is mainly used for XG production in industries [7,12].

The production of XG is affected by various factors such as the type of carbon source (glucose and/or saccharose), microorganism, and the operating conditions (continuous or batch). To ensure the high yield and better-quality production of XG, it is necessary to evaluate the settings of a bioreactor, concentration of nutrients in the growth medium, time of agitation, optimal pH and temperature, aeration, and fermentation time [7,12].

**Figure 1:**
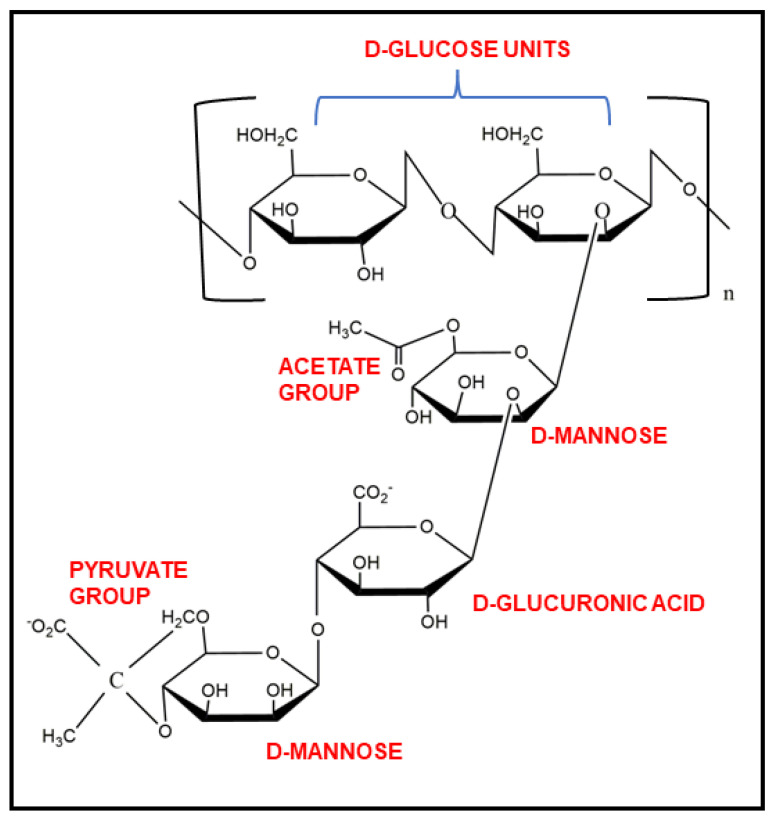
Chemical structure of xanthan gum and its functional groups.

Xanthan gum is a dry, yellow-white coloured powder of high molecular weight (1 × 10^6^ to 20 × 10^6^ Da) exopolysaccharide. It is highly water soluble in both hot and cold conditions due to its polyelectrolyte behaviour [13], though constant mixing is needed to prevent agglomeration. Water-dispersed XG shows viscous solution even at very low concentrations [14]. XG shows high stability in acidic as well as alkaline mediums. It is a cellulose derivative and, thus, is a biodegradable gum in nature. It has cellobiose as a repeating unit in its backbone, and the side chain is composed of D-mannose, D-glucuronic acid (β-1,2), and D-mannose (β-1,4), a trisaccharide, linked to each other by α-1,3 linkages as shown in Figure 1. Approximately one-half of the terminal D-mannose holds a pyruvic acid residue linked by the keto group at the 4th and 6th positions, with an indefinite distribution. The non-terminal D-mannose is connected with the core backbone chain and contains an acetyl group at the O-6 position. The relative composition of pyruvyl and acetyl residues in XG chains mostly depends on the *Xanthomonas* strain used for its production [7].

**Figure 2:**
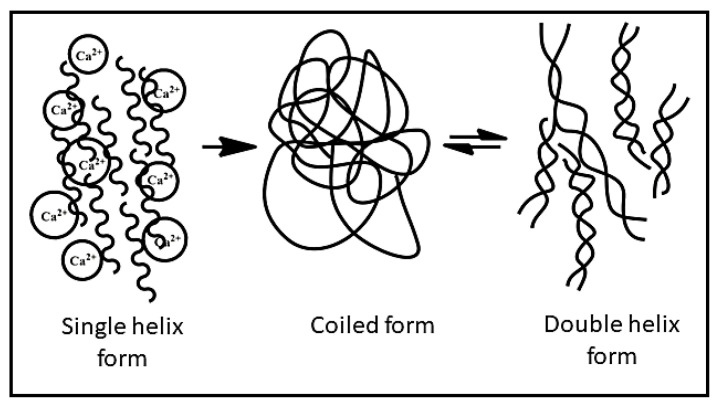
The conformational changes in xanthan gum.

XG behaves as a polyanion at a pH above 4.5 because of the deprotonation of O-acetyl and pyruvyl residues. After extraction from the broth, XG is present in a single helix form stabilised by already present Ca^2+^ ions. The helix form can be converted to coils irreversibly. The coiled conformation of XG can be converted into a double-helical structure reversibly, as illustrated in Figure 2. These conformational changes in the XG structure can be controlled by varying the ionic strength and temperature [7].

XG exhibits pseudoplastic behaviour, which can be modified by varying temperature and shear rate, making XG useful in the food industry as a thickener, stabiliser, and suspending agent for food [14]. It shows a decrease in viscosity at high temperatures and remains stable when solubilised. XG also has high thermal stability, which prevents its degradation or hydrolysis, making it unique and more advantageous than other hydrophilic polysaccharides. The viscosity of the solutions containing XG is affected by various factors such as pH, the concentration of buffer solution, and the concentration of XG in the solution. Deacetylation of XG occurs in extreme alkaline pH (pH 9 or above) and forms a gel at higher pH (>pH 10) [15]. Table 1 shows the physicochemical properties of commercially available XG.

## 3. Important Properties of XG for Designing Drug Delivery Systems

XG is essentially used as a carrier system for the delivery of drugs, proteins, and genes. The important characteristics of XG for this purpose are (i) high stability at low pH, which helps in the protection of the drug in gastric fluid from degradation, and (ii) the rate of drug release can be controlled by changing the pH of the release medium and ionic strength. XG has been used in the form of tablets, hydrogels, microspheres, and mucoadhesive gels for the sustained and controlled release of drugs [16]. For a targeted release of the drug, a controlled release is anticipated using different mechanisms. Such systems ensure an increase in the bioavailability of drugs at the targeted site and avoid drug loss in gastric degradation [17]. Xanthan gum acts as an acknowledged excipient for controlled and sustained-release formulations. Combining XG with other polysaccharides improves and achieves controlled release of the drugs. Even a small amount of XG improves the drug release and provides zero-order release kinetics. XG used as a mucoadhesive patch provided a controlled release of nicotine over a 10 h period with burst release of the drug initially [18]. Floating tablets consisting of a mixture of guar gum and XG encapsulating rosiglitazone maleate showed sustained release (98%) for up to 12 h [19]. For the controlled release of pentoxifylline (hydrophilic drug), XG-based tablets were prepared, which showed a decrease in drug release with an increase in polymer concentration [20]. pH-responsive interpenetrating network microspheres were prepared for the controlled delivery of diclofenac sodium. XG and polyvinyl alcohol (PVA) were used for the development of microspheres, and the ratio of polymers and extent of crosslinking greatly affected the release pattern of the drug [21].

### 3.1. Mucoadhesive Properties

To enhance the retention time of the drug and increase its therapeutic activity, mucoadhesion is a significant approach that holds the carrier to the biological site by interfacial forces. Mucoadhesive property is important for formulation such as ophthalmic, skin, gastro-retentive, and nasal formulations. Altering the physicochemical properties (concentration, molecular structure) of the polymer has a significant impact on the mucoadhesive property of the polymer [22]. Xanthan gum shows inherent mucoadhesive properties and is used for the synthesis of various drug delivery systems. Mucoadhesive buccal patches using XG were synthesised for the delivery of zolmitriptan. The drug permeability was increased by 3.3-fold [23]. The nanoemulgel system was prepared using XG as an anionic mucoadhesive polymer for the delivery of carbamazepine via olfactory mucosa to treat epilepsy. The results showed a decrease in peripheral actions of the drug [24]. Chitosan-XG-based mucoadhesive polyelectrolyte nasal insert was prepared for the delivery of promethazine hydrochloride (HCl). The surge in the concentration of XG delayed the release of the drug and showed 89% of mucoadhesion with nasal mucosa [25].

### 3.2. In Situ Gelling and Rheological Properties

Polymeric formulations are known as “in situ gelling systems” and show a transition from their sol state before entering the body to their gel state once inside. The pH shift, temperature modification, solvent exchange, UV radiation, and the presence of certain ions or molecules are only a few of the stimuli that can cause the sol-gel transition [26]. XG is present in the dry state; however, when it encounters water, it turns into the hydrated glassy state and, further, with an increase in swelling degree, changes to a more porous and rubbery state. The release of the drug can be dependent on the pH of the medium because there is a conformational change in XG with changes in ionic strength and pH [18].

At low ionic strength or high temperature, the XG chains are assumed to be present in coiled conformation, whereas, at high ionic strength or low temperature, the conformation of XG chains changes to the helical form. Hydrogen bonds stabilise the helical conformation of the gum, while electrostatic repulsion between carboxylate groups destabilises the structure [27]. XG already has calcium ions present, which destabilises the helical conformation. Further addition of Ca^2+^ ions in stoichiometric equivalence leads it to a gel-like state. However, adding Ca^2+^ ions more than its stoichiometric equivalence partially replaces the carboxyl groups and decreases the network formation and degree of complexation [28].

The low content of the pyruvyl group lowers the viscosity, and the high pyruvyl content increases the viscosity of the gel. Higher acetyl content decreases the gelling capacity of xanthan gum in an aqueous solution [29,30,31]. The viscosity of the XG solution greatly depends on various factors, including pH, the concentration of buffer solutions, and the concentration of XG [31].

## 4. Designing Various Forms of Delivery Systems Using XG

In the pharmaceutical and medical fields, natural polymers are preferred over semi-synthetic polymers as drug delivery systems because of properties such as low toxicity, low cost, high availability, and non-irritant nature. However, there are several drawbacks to using natural polysaccharides as an excipient, such as a wide variation, microbial contamination, decreasing viscosity with long-term storage, unsuitable mechanical properties, and uncontrollable rate of hydration. To overcome these disadvantages, chemical modification to the pure polysaccharides is carried out [32]. XG is used as a drug-loading excipient in many forms.

### 4.1. Matrix Systems

The regulated release of drugs is accomplished using biopolymeric matrix systems. Polymeric materials which swell in the biological fluids or water form matrix systems. The drug is evenly distributed across the matrix of polymeric material before being compressed to form a tablet. The drug that has been dissolved or dispersed follows a regulated drug release pattern. XG is used as a matrix tablet for the controlled release of the drug [16]. XG is used in tablets both as a binder and disintegrant. The polymer’s ability to swell facilitates tablet breakdown and speeds up pharmacological absorption [33]. Graft copolymerisation of acrylamide onto XG was done for the delivery of antihypertensive drugs (atenolol and carvedilol). The release patterns were compared to that of the commercially available formulations, and a non-Fickian trend of transportation was followed [34].

### 4.2. Films

In addition to tablets, xanthan gum-based films have been thoroughly researched and used. Due to their adaptability, films can be used as a dosage form for several purposes (buccal, oral, sublingual, ophthalmic, transdermal, etc.). A drug delivery film is typically composed of a thin, flexible polymeric layer that may or may not contain a plasticiser. Because of these qualities, they are less obstructive for the patient, making them a more desirable mode of administration [35,36]. Mouth-dissolving films were prepared using XG for the rapid absorption of glibenclamide to treat diabetes. The solvent casting method was for the synthesis of films. The films showed optimum mechanical strength with instant drug release and rapid dissolution rate [37]. For the delivery of zolmitriptan, a bilayered mucoadhesive buccal film was prepared using XG, hydroxypropyl methylcellulose (HPMC)-E15, and polyvinyl alcohol (PVA). Instant drug release occurred in the initial 15 min, and further sustained release of the drug was observed. Upon the addition of dimethyl sulfoxide (DMSO), the permeability of the drug increased 3.2-fold without any damage to the buccal mucosa [23]. The gum’s incorporation in the films reduced the moisture content and water vapour permeability in addition to the enhancement of mechanical and thermal qualities. In some cases, films are not an appropriate dosage form since they require a thick platform. The use of hydrogels in this situation might be an alternative.

### 4.3. Hydrogels

Hydrophilic polymer chains in an aqueous microenvironment crosslink to produce materials called hydrogels. Gelation is accomplished by a variety of methods, including covalent chemical cross-linking, electrostatic interactions, and physical cross-linking [38]. These polymeric networks’ physicochemical characteristics and mechanical stiffness are determined by the procedure and cross-linking grade [39]. Hydrogels are useful for encapsulating pharmaceuticals because of their high hydrophilicity (ability to retain water from 70% to 99%), which preserves the structural integrity of the drugs and lowers the risk of enzymatic denaturation once inside the body [40]. Several studies on XG-based hydrogels for the delivery of drugs have been carried out. XG hydrogels provide a controlled release of drugs. Succinic anhydride-modified xanthan gum has been used to prepare ionic strength-sensitive hydrogel for the delivery of gentamicin. Initially, a rapid release of gentamicin was observed with increased ionic strength of the release medium. After that, a sustained release of the drug was observed because of the strong interaction between polymer molecules, forming a tight network [41]. To increase the bioavailability and contact time between the drug and the precorneal membrane, the in-situ hydrogel was synthesised and used to deliver the drug. XG and sodium alginate were used as a mucoadhesive polymer, and poloxamer 407 and poloxamer 188 were used to encapsulate moxifloxacin hydrochloride. The formulations prepared were transparent, uniform in consistency with good spreadability, and with optimum bioadhesion properties [42].

## 5. XG-Based Systems for the Delivery of Drugs

### 5.1. Delivery of Anti-Microbial Drugs

Infections brought on by various microorganisms pose a serious hazard to human health. The infections caused by microorganisms are treated using three different categories of drugs—antiviral, antibacterial, and antifungal [43]. These antimicrobial drugs have proven to be effective against a wide range of infectious diseases. However, patients’ options for therapy are constrained since bacterial resistance develops faster than the development of antibacterial drugs. The number of multi-drug-resistant organisms is the reason behind mortality and morbidity worldwide. Nanoparticles have shown synergy when used with the best antibiotics, and they could help to control bacterial resistance development. XG is used as an anti-microbial drug carrier that exhibits a controlled and sustained release of drugs. XG also helps in the formation of different hydrogels, tablets, and mucoadhesive patches for the delivery of antibiotics. To improve the residence time and bioavailability of besifloxacin, in situ ocular gels were prepared using XG, sodium alginate, and ethyl cellulose in different ratios. Besifloxacin is a fluoroquinolone antibiotic that inhibits the DNA gyrase (topoisomerase II and IV) and is used against bacterial conjunctivitis. Different formulations were prepared and evaluated for pharmacopoeial specifications. It was observed that the optimised formulation was transparent, clear with high gelling capacity, non-irritant, and showed sustained drug release (for 8 h) with high drug content capacity (96.2–98.6%) [44].

Similarly, in situ ocular gel was synthesised using a combination of polymers: XG, hydroxypropyl methylcellulose (HPMC), and sodium alginate in different ratios. This in situ gel was employed for the delivery of flucytosine, a semisynthetic macrolide antibiotic for the treatment of trachoma (caused by *Chlamydia trachomatis*). The synthesised gel aimed to achieve prolonged pre-corneal retention time and improved bioavailability of the drug in comparison to the conventional dosage form. The drug-loaded formulation showed high drug content (95.2–98.6%), sustained drug release (up to 8 h), and stability at both room and higher temperatures [45]. In a study, a floating low-density tablet formulation of stavudine was prepared using different polymers: XG, HPMC K15, and HPMC K100M for HIV treatment. The formulation of 80 mg tablet with a flat face, 9 mm diameter, was prepared to increase the half-life of stavudine in gastric fluid. Sustained release of the antiviral drug stavudine was achieved with all the pre-compression and post-compression characteristics [46].

**Figure 3:**
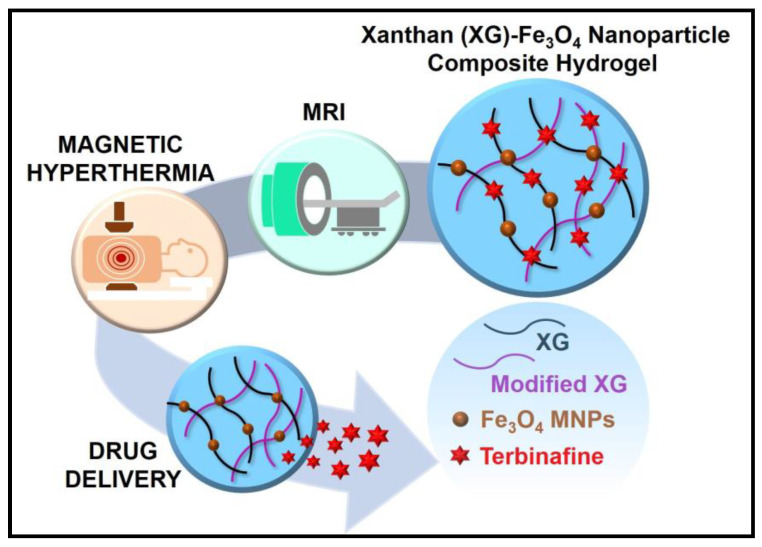
Xanthan gum-Fe_3_O_4_ nanoparticle composite hydrogel for the theranostics application [47]. Reproduced from ref. [47]. Copyright 2021 American Chemical Society.

Modification in the XG structure is beneficial for the delivery of drugs. XG was modified and conjugated with magnetic nanoparticles. Implantable xanthan gum/Fe_3_O_4_—magnetic nanoparticle composite hydrogel was synthesised for a non-invasive imaging study as shown in Figure 3.

The modified polymeric matrix encapsulated terbinafine (hydrophilic drug) and magnetic iron oxide. It showed rheological shear-thinning and enhanced swelling properties. The hydrogel also demonstrates thermally induced controlled drug delivery (3-fold) by magnetic hyperthermia and high efficacy compared to the pure drug for antifungal properties. The incorporation of Fe_3_O_4_ nanoparticles helped the hydrogel in functioning as non-invasive monitoring by magnetic resonance imaging (MRI). Magnetic hydrogels of different concentrations were compatible with normal human dermal fibroblasts and opened a new outlook in the biomedical application of XG [47].

In a study, two polymers, Chitosan and XG, were cross-linked with 2-acrylamide-2-methylpropane sulfonic acid (monomer). The polymeric network was synthesised using the free radical polymerisation method for the delivery of acyclovir, which is a highly selective antiviral drug, for the treatment of prophylaxis and *Herpes simplex* virus infections. The system showed pH-dependent drug release and swelling behaviour. It was observed that the hydrogel had a porous structure and was thermally stable [48].

### 5.2. Delivery of Chemotherapeutic Agent

XG is decomposed in the presence of colon enzymes rather than digested in the stomach or small intestine of humans. XG matrices are used to deliver the drug to the colon while protecting it from the stomach and small intestine environments. When they enter the colon, they are reduced into smaller monosaccharides by the anaerobic microflora present in the colon and either used as an energy source by the bacteria or broken down by an enzyme [49]. Microspheres and hydrogels were synthesised using XG to be targeted against colon cancer. α-Linolenic acid and docosahexaenoic acid were loaded in the formulations and were evaluated for their antioxidant and antineoplastic activities. The loaded formulations: microspheres encapsulated α-linolenic acid efficiently, whereas hydrogels were loaded with both the polyunsaturated fatty acids. The antioxidant activity and anti-neoplastic activity of α-linolenic acid were increased when loaded into the XG-based nanoformulations, whereas the activity of docosahexaenoic acid remained the same when tested against colon cancer cells [50]. 5-Fluorouracil is a chemotherapeutic drug used in the treatment of various cancers. The delivery of this drug causes serious side effects in the body, such as mucositis, alteration in the normal microflora environment of the colon, and diarrhoea [51]. For the target-specific treatment of colon cancer, 5-fluorouracil was encapsulated within XG-poly(-vinylpyrrolidone-co-poly) (PVP)—acrylic acid-based hydrogels. The pH-sensitive hydrogel was prepared using different concentrations of polymer, monomer, and crosslinker. The gel fraction was directly proportional to the concentration of polymer, monomer, and crosslinker, whereas the swelling capacity decreased with the increased concentration of polymer and crosslinker. It showed stability in a wide range of pH media and maximum swelling in a basic medium, confirming its use as a pH-responsive hydrogel. In rabbit models, acute oral toxicity studies were performed, demonstrating that the hydrogels were non-toxic. It was inferred that prepared pH-sensitive hydrogels were ideal for the delivery of 5-fluorouracil to the colon for sustained release [52]. Treatment of colorectal cancer by encapsulating 5-fluorouracil prebiotics and probiotics was an approach taken by Singh et al. [51]. The system with three components was taken: drug-loaded nanoparticles coated with prebiotics (XG, guar gum) and probiotics (*Bifidobacterium bifidum*). XG and guar gum were used to enhance the delivery of 5-fluorouracil in the colon and protect it from gastric juices. In vitro and in vivo studies showed intact microflora of the intestine, which fulfils the aim of the research of overcoming the side effects of 5-fluorouracil by co-administration of probiotics (*B. bifidum*) [51]. For the delivery of a well-known chemotherapeutic agent, curcumin, XG-chitosan nanofibers were prepared. The developed nanofibers were stable at pH 6.5 and pH 7.4. When tested against Caco-2 human colon cancer cell line, the loaded curcumin showed more efficacy than the pure drug. Further, the permeability of curcumin was increased 3.4-fold in the presence of XG-chitosan nanofibers [53].

pH-responsive nanoparticles [54] and nanogels [55] of XG were prepared for the delivery of doxorubicin, an anticancer drug used for the treatment of different cancers. The nanoparticles (110–180 nm) showed drug loading of 15.2% and pH-sensitive drug release. The nanoparticles were found to be biocompatible [54]. The nanogels were synthesised by amidation of xanthan gum with cysteamine tetra-hydrazide. The nanogels showed good pH sensitivity due to the presence of disulfide bonds. The disulfide bonds could be reduced by glutathione, causing drug release from the nanogels. Nanogels were also nontoxic and biocompatible, proving to be an ideal carrier for the delivery of chemotherapeutic drugs [55]. Gold nanoparticles were synthesised for the delivery of doxorubicin, and XG was used to stabilise the nanoparticles and as a reducing agent.

The XG stabilised gold nanoparticles were obtained with 5 mL of 1.5 mg/mL XG solution. The nanoformulation was stable under different pH conditions (pH 5–9). In vitro studies showed that nanoparticles coated with xanthan gum had a 3-fold increase in efficacy of the drug against the A549 human lung cancer cell line [56].

Similarly, thermos-responsive gold nanoparticles were synthesised and coated with carboxymethyl xanthan gum by microwave irradiation method. The release of the doxorubicin was extensive at acidic pH and negligible at physiological pH. The in vitro studies of doxorubicin-loaded nanoparticles showed a 4.6-fold increase in the anticancer activity of the drug compared to the free drug [57].

### 5.3. Delivery of Anti-Diabetic Drugs

Diabetes mellitus (DM) has emerged during the past few decades as one of the primary fatalities on a global scale [58]. Although many hypoglycaemic drugs are frequently used in diabetes control, a cure for diabetes is still a long way off because of the various inherent flaws and negative side effects of the drugs [59]. Due to proteolytic degradation and chemical instability in a severe pH environment, the necessary optimal drug concentration cannot reach focal regions. Additionally, substantial fluctuations in glucose that cause severe hypoglycaemia cannot be addressed with traditional dosing forms [60]. Other problems with pharmacological therapy, such as poor drug absorption, a short half-life, poor solubility, poor bioavailability, and negative effects on other organs, make treatment difficult [61]. XG owing to its various benefits is used for the delivery of anti-diabetic drugs to improve its bioavailability and solubility. Glimepiride is a hypoglycaemic drug with low solubility and bioavailability. It is extensively used in the treatment of diabetes (type 2). Transdermal delivery of the drug is desired with high permeation and increased solubility. Topical nanoemulgel was formulated using clove oil, PEG-400, and Tween-80 and further gelled with xanthan gum (3% *w/w*). The formulation significantly increased the permeation and bioavailability of a drug [62]. Glibenclamide is also a hypoglycaemic drug and is used to manage type 2 diabetes. It tends to pass through gastrointestinal mucosa. XG-based mouth-dissolving films were synthesised, and glibenclamide was loaded onto them. The films were formed for the rapid release of the drug and to increase its bioavailability in the body. The optimised formulation showed drug loading of 96% and decomposed within 40 s. Stability studies proved that the formulation was stable even in extreme conditions [37]. Repaglinide is an antidiabetic drug used to manage diabetes in humans. A mucoadhesive buccal tablet of the drug was prepared for the prolonged release of the drug in the system. XG and HPMC K100M polymers were used for the assessment of bioadhesion and release of the drug. The drug loading was 96.55%, and the release of the drug followed non-Fickian diffusion. The drug release was directly proportional to the concentration of both the polymers used. Stability studies showed that the tablets were stable for up to 2 months without any decrease in the drug content [63]. Tolbutamide has less bioavailability, and thus its efficacy in the body decreases. To enhance the bioavailability of the drug, xanthan gum-stabilized gold nanoparticles were synthesised. The loaded formulation showed high loading efficiency without changing the structure of the drug. In animal studies, insulin secretion was found to be higher in the mice treated with the developed formulation in comparison to the mice treated with pure drug solution [64].

### 5.4. Delivery of Drugs for the Treatment of Cardiovascular Diseases

**Figure 4:**
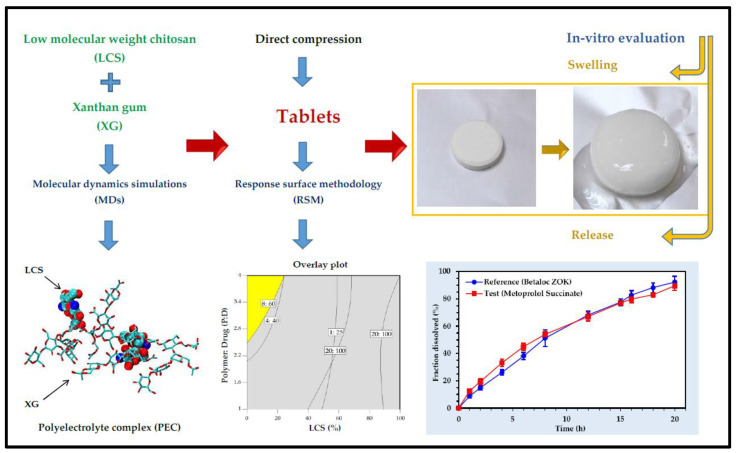
Development of xanthan gum-chitosan directly compressed tablets for the controlled release of metoprolol succinate [69]. Reproduced from ref. [69]. Copyright 2020 American Chemical Society.

For the treatment of cardiovascular diseases, XG-based carrier systems were developed. Verapamil hydrochloride is a calcium channel blocker and is used to lower blood pressure in the body. To achieve a controlled and sustained release of the drug, a matrix was synthesised using different polymers. XG, HPMC, and corn starch were used to delay drug release. Using carboxymethylation, different formulations were made of the drug with different polymers. XG, among all, significantly reduced the surge in the release of a drug. The drug release was dependent upon the concentration of the polymer [65]. Furthermore, for the sustained release of propranolol hydrochloride, tablets were produced using XG and chitosan. The drug release pattern was observed in both of the polymers individually and in a combination of both (1:1) as a matrix, and with the addition of lactose (75% *w/w*), the mixture of both polymers prolonged the time of drug released from the matrix in pH 1.2 [66]. For the delivery of the drug, carvedilol, transdermal patches were synthesised to increase its bioavailability and to protect the drug loss in the first pass event. Different formulations were prepared using XG, HPMC K4M, and sodium alginate in different ratios. Formulations containing xanthan gum showed more than 85% of drug release within 12 h and followed Korsmeyer–Peppas’ kinetics during drug release [67]. To increase the bioavailability and for sustained release of diltiazem HCl, different membranes with high and low molecular weight xanthan gum and poly (-vinyl alcohol) were synthesised. The mechanical strength of a high molecular weight polymer-based membrane was enhanced compared to a low molecular weight polymer-based membrane, whereas the physical properties were similar. Thinner membranes showed high encapsulation, whereas the thicker membrane showed more sustained and slow release of the drug [68]. In a study, a controlled-release pharmaceutical solid dosage form including metoprolol succinate, low molecular weight chitosan, and xanthan gum was developed and evaluated (Figure 4) [69].

Drug release and computational studies revealed low molecular weight chitosan performs better than high molecular wright chitosan in controlling the release of the drug when directly compressed with XG. Metoprolol succinate was found to be localized more in the core of the tablets in the beginning stages of dissolution because of film formation between XG and low molecular weight chitosan, which prevents the penetration of excessive water in the tablet matrix. The film begins to disintegrate or erode in the final stages of the breakdown process, enabling complete tablet hydration and a consistent drug distribution in the swelling tablet [69].

### 5.5. Anti-Spasmodic Drug Delivery

Irritable bowel syndrome (IBS) is a condition characterised by cramping, stomach pain, bloating, constipation, and diarrhoea. IBS affects women more than males, with a female-to-male ratio of 2–2.5:1 among those who seek medical care [70]. Anti-spasmodic drugs serve to prevent or treat painful cramping spasms in the intestines by relaxing the smooth muscles of the gut [71]. In a study, a floating tablet using XG and sodium alginate was prepared by an effervescent method [72]. Citric acid and sodium bicarbonate were used as gas-generating agents. Fenoverine is an antispasmodic medication used to treat muscle spasms, stomach cramps, and irritable bowel syndrome-related abdominal pain. Pre-compression studies were carried out, and for the directly compressed tablets, post-compression studies were done. The polymers enhanced the viscosity of the formulation in the dissolution medium and gave a sustained release of the drug efficiently for up to 12 h. Of the different formulations prepared, the formulation having 15% XG was determined to be the ideal formulation with 99.6% drug release in 12 h. It also showed a better swelling ratio with required drug release kinetics and floating behaviour. The floating tablets were ideal for the delivery of the drug and increased its bioavailability and sustained release in the system [72].

### 5.6. Delivery of Drugs for the Treatment of Inflammation, Rheumatoid Arthritis, and Gout

Rheumatoid arthritis and gout are common types of inflammatory arthropathy [73]. Non-steroidal anti-inflammatory drugs are used for the treatment of inflammatory disorders but have serious adverse effects, such as kidney damage [74]. To increase the efficacy of anti-inflammatory drugs and reduce their side effects in renal diseases, XG is used as a carrier system. It reduces the need for repetitive dosage and provides sustained release of the drug. Diclofenac potassium is a non-steroidal anti-inflammatory drug, and research was carried out to find the adsorption of graphene oxide nanoparticles on the drug [75]. To increase the adsorption of the graphene oxide, the co-polymeric hydrogel was prepared. XG and poly-acrylic acid were used to achieve sustained release of the drug by forming composite hydrogel with drug-loaded graphene oxide. The swelling ratio and drug release certainly increased with the increase in the pH. The drug-loaded composite hydrogel was pH-responsive. In vivo studies concluded that the bioavailability of the drug increased with an increase in the concentration of composite hydrogel [75]. Similarly, for the delivery of the same drug, double-walled carbon nanotubes enclosed with xanthan gum-based hydrogels were formed for the improvement of drug release. Carboxymethylation of the XG was carried out, and an increase in viscoelasticity was observed due to the presence of XG in the system. The encapsulation efficiency of the diclofenac was increased to 75%, and the sustained release of the drug was observed due to the presence of nanotubes. The composite was stable in extreme conditions similar to that which may be present on the skin [76]. For the delivery of lornoxicam-loaded thiolated XG as a topical gel formulation, it was synthesised by the microwave esterification reaction. The thiolation of the xanthan gum was carried out using thioglycolic acid [32]. The physicochemical properties of the gel formed were ideal, and the drug content in the thiolated gel was 98.4–99.7%. The in vitro drug release studies showed sustained release for up to 24 h. The thiolated gel was stable over a wide range of temperatures and was found to be ideal for topical drug delivery in the treatment of inflammation and pain [32].

Mucoadhesive buccal tablets were prepared using different concentrations of XG and *Vigna mungo* for the treatment of gout. The tablets were used to increase the bioavailability of febuxostat at the mucosal site for a prolonged time; the sustained release of the drug was 8 h. The tablets were stable at room temperature and showed good mucoadhesive properties [77].

To avoid an acidic environment and drug degradation in the stomach, XG-chitosan microparticles with pH-sensitive Eudragit^®^-L were prepared for colon-targeted delivery of the drug. Quercetin is a flavonoid with low bioavailability showing anti-inflammatory and antioxidant properties [78]. The system was prepared to increase oral drug bioavailability and enhance its release in the intestine for inflammatory-based disorders. The microparticles were loaded with quercetin, and the release was controlled by non-Fickian diffusion of the drug. The microparticles were compressed and converted into tablets with a coating of Eudragit [78]. To evaluate the effect of changing concentrations of hydrophilic and hydrophobic polymers on the sustained release of metoclopramide encapsulated in matrix tablets. The matrix tablets were prepared using XG and ethyl cellulose together and individually. Pre-compression and post-compression parameters of the tablets were found to be under the limit. The tablets prepared using XG and XG-ethyl cellulose both showed sustained drug release for up to 12 h. Drug release kinetics followed the Korsmeyer–Peppas model [79]. For the treatment of colitis, curcumin-loaded pH-responsive polyacrylamide-grafted-xanthan gum nanoparticles were prepared. The drug release was less (~8%) at acidic pH 1.2 in comparison to the drug released (~35%) at pH 7.2. These results indicated the pH-responsive nature of the formulation. Further, the drug release from the formulation was ~65% in rat caecal pH 6.8, suggesting microflora-dependent drug release. In in vivo studies, drug-loaded nanoparticles demonstrated a greater reduction in colonic inflammation, better prevention of weight loss, and more decrease in myeloperoxidase and nitrite levels than pure drug [80].

### 5.7. Delivery of Immunosuppressive Drugs

To assure therapeutic outcomes, immunosuppressant drugs are typically administered via conventional techniques that frequently demand high doses of the drugs, although this can lead to harmful side effects such as infections, cancers, and specific side effects in pregnancy [81,82]. Synthesis of formulations using XG carrying the optimum amount of drug to be effective at the target site was achieved by Sadeq et al. [83]. Ketotifen fumarate is an antihistamine (selectively blocks histamine H1 receptors) and is used in conjunctivitis and rhinitis. It has low solubility and belongs to Biopharmaceutical Classification System (BCS) class II drugs. To enhance its bioavailability in ocular tissues, it was loaded into in situ ocular gel for the treatment of eye infections. pH-sensitive ocular gels were prepared for better efficacy than the formulation present in the market. Different concentrations of polymers: xanthan gum, Carbopol, and gellan gum, were taken to form different formulations. Amongst the optimised formulations, formulation with 0.75% Carbopol and 0.3% XG was found to be optimum. It had a drug encapsulation efficiency of 99.74%, gel-forming strength in 46.6 s, and drug release of up to 8 h [83]. These findings demonstrate the impact of polymer combinations; it was observed that as XG or gellan gum concentrations rose, the percentage of the drug release reduced. This is due to increased gel strength and decreasing the size and quantity of gel structure channels, which limit the movement of the drug molecule and minimise dissolution media penetration, delaying the time at which the drug is released [83,84]. To enhance the bioavailability of the drug, Tacrolimus microbeads were synthesised. A highly effective immunosuppressive drug with demonstrated effectiveness in both in vivo and in vitro studies, Tacrolimus is also a calcineurin inhibitor. Varying concentrations of XG and sodium alginate were used to form microbeads. The sodium alginate was taken as the main polymer but to increase the drug-loading efficiency of microbeads, xanthan gum was added to the formulation. Using different concentrations of crosslinkers also affected drug release. Furthermore, the drug release is dependent on the concentration of the polymer and coating polymer. It was suggested that this system could be used as an alternative and cost-effective system for the delivery of drugs [85].

### 5.8. Delivery of Drugs for the Treatment of Skin Diseases

For topical delivery of a drug, the skin itself serves as a natural shield against particle penetration. It also provides a potential method for administering medications, particularly to damaged skin and through the apertures of hair follicles. Recent advancements in dermatological treatment may be made by improving the dermal localisation of bioactive components into the damaged skin region. These nanocarriers can concentrate at the target site after application by passing through the stratum corneum into viable skin [86]. Xanthan gum-based nanocarrier systems help in the efficient delivery of drugs for the treatment of various skin disorders. Pycnogenol is a French maritime pine bark extract that is a concentrate of phenolic compounds. It shows several biological activities such as anti-inflammatory, anti-oxidant, and anti-bacterial and is used in the treatment of open wounds. For the enhanced release of the extract, a bioadhesive film using sodium alginate and XG was prepared in different ratios [87]. The film showed suitable mechanical properties such as high deformability, suggesting easy adaptability to any type of surface. The film shows high bioadhesion on the skin and absorption of exudates from the open wound. In vitro studies showed that drug-loaded films can inhibit the growth of bacterial strains, including *S. pyogenes, S. aureus,* and *E. faecalis*. Together with the help of sustained release of the drug, it was attributed that the formulation needs to be applied once a day, and it helps in the growth of keratinocytes, thus, healing the wound [87].

### 5.9. Delivery of Drugs for the Treatment of Central Nervous System-Related Disorders

The consequences of so-called modernised living include increased stress, mental disorders, and psychological misbehaviours due to humans’ increasing desire and avarice. Every year, a wide range of mental illnesses claim millions of victims, and the need for treatment is only expanding. The blood-brain barrier and the blood-cerebrospinal fluid barrier restrict drug access to the central nervous system (CNS). These barriers can be overcome or bypassed by biomaterials, allowing for the controlled administration of medications into the CNS [88]. Quetiapine fumarate is a typical antipsychotic medication and is used in the management and treatment of psychosis, bipolar disorder, and other neuropsychological or affective disorders. To enhance the bioavailability of quetiapine fumarate and protect it from first-pass metabolism, mucoadhesive matrix tablets were prepared. The matrix tablets consisted of a mixture of polymers (xanthan gum, Carbopol 934P, HPMC K4M, polyvinyl pyrrolidone K30) and showed a sustained release of drug (96% in 10 h) along with ~77% permeation across the mucus membrane [89]. Furthermore, to increase the bioavailability of the drug and surface characteristics in the solid state, tablets—a blend of drugs with the XG and tragacanth gum were synthesised [90]. The results presented that the type of natural gum and its structure greatly affected drug release. XG exhibited a swelling mechanism for the controlled release of the drug. The binary tablets formed with XG showed pH-responsive drug release, fitting Higuchi and Korsmeyer–Peppas models [90].

Another drug, zolmitriptan, is a triptan that is used for the cure of migraine attacks with/without aura and clusters. To enhance the sustained release of this drug, oral tablets were synthesised for the treatment of migraine. Preparation of the tablets was carried out using xanthan gum, guar gum, and karaya gum by direct compression method. Formulations containing xanthan gum showed an increase in sustained release of the drug with the increase in the concentration of polymer [91]. For the treatment of Alzheimer’s Disease, the administration of resveratrol-loaded lipid nano formulation via nasal route was prepared. The lipid carrier was incorporated in xanthan gum and gellan gum for in situ gel formation. The optimisation of in situ gel resulted in 5-fold higher permeation in the nasal mucosa when compared to pure drug suspension-based gel. The drug-loaded formulation was evaluated using the scopolamine-induced amnesia model in mice, and improvement in memory function was observed [92].

### 5.10. Delivery of Drugs for the Treatment of Obesity

Orlistat is a lipase inhibitor that blocks the absorption of fat from the body by blocking certain enzymes responsible for the breakdown of fats. It is used in the treatment of obesity and is taken with a low-calorie diet. The drug has several side effects, such as stool of fatty and oily consistency. To reduce side effects, an oral dosage formulation of orlistat was developed. Xanthan gum was used as an oil-trapping agent. Mini-tablets of orlistat (10 in no.) were co-administered with mini-tablets of XG (40 in no.). The drug release of the optimised mini-tablets was compared to commercial tablets available; both showed similar drug release profiles [93]. The formulation had a large amount of XG, but it has already been proven that XG is safe in food additives (FDA-approved). Comparative in vivo studies were performed between optimised mini-tablets and commercial forms of drug using Sprague-Dawley rats. The faeces of the treated rats were analysed, and the faeces were significantly less oily and fatty when treated with the optimised formulation of orlistat and xanthan gum. As a matter of patient compliance, optimised mini-tablets were easy to swallow. The overall conclusion was that the mini-tablets formed using XG as an oil-reducing agent might help in the development of many other delivery systems for anti-obesity drugs to reduce their side effects [93].

### 5.11. Delivery of Drugs for the Treatment of Glaucoma

Acetazolamide blocks carbonic anhydrase, which also reduces the build-up of certain fluids in the body. It is used to treat glaucoma, in which there is fluid build-up in the eye, creating pressure. To improve the efficacy of the drug and achieve its sustained release, the drug was encapsulated in a nanoemulsion-based in situ gel [94]. Different nanoemulsion formulations were prepared using different surfactants. For pH-responsive in situ gel formation, pure gellan gum and in combination with HPMC, xanthan gum, and Carbopol were used. The in situ gels showed improved sustained release than nanoemulsion. A combination of XG with gellan gum proved to be the ideal formulation, with increased mucoadhesive properties and a relative decrease in ocular pressure compared to commercial oral tablets and eye drops [94]. Dorzolamide hydrochloride is also used to treat glaucoma. A mucoadhesive in situ gelling formulation was prepared for the treatment via the ocular route. XG and poly acrylic acid were used in combination as well as individually for the synthesis of in situ gel. Formulation with 0.2% *w/v* XG showed 90.84% of drug release for 12 h. The drug release was also analysed using goat’s cornea, and the formulation showed similar release kinetics to the in vitro release for up to 9 h [95].

### 5.12. Delivery of Drugs for the Treatment of Pulmonary Diseases

For the treatment of respiratory conditions associated with lung cancer, asthma, and chronic obstructive pulmonary disorders, pulmonary medication delivery allows targeted therapy. However, the rapid clearance mechanism, drug deposition, and instability mechanism present problems with this method. The effectiveness of pulmonary delivery systems is further influenced by additional elements such as the kind of inhaler device, patient compatibility, consistent device delivery, and inhaler technique [96]. In order to address these problems, nanotechnology has been used in pulmonary delivery systems, targeting the direct intake of aerosols into the respiratory epithelium and epithelial cells. The architecture of the aerosol formulation can be altered to offer either rapid absorption or prolonged retention depending on the desired drug release characteristics [97]. For the controlled release of theophylline, interpenetrating polymer network-based hydrogel beads were synthesised.

Sodium carboxymethyl xanthan and casein were crosslinked with aluminium chloride and glutaraldehyde to synthesise the hydrogel beads. Theophylline belongs to the class of drugs known as xanthines. It is a bronchodilator and is used to manage symptoms of Chronic Obstructive Pulmonary Disease (COPD), asthma, and other various lung conditions due to reversible airflow obstruction. Dissolution studies and drug release were carried out in both acidic and alkaline mediums, and delayed release of the drug was observed [98]. Furthermore, to control the dose dumping of theophylline, XG was used as a rate-controlling gum to synthesise alcohol-resistant mini-tablets and matrix tablets. It increased the solubility of the drug by 2.8 times in 40% alcohol. At lower concentrations of XG with large particles, the drug release was observed to be faster when compared to the higher concentration of polymer with finer particles (<75 µm) in dissolution studies [99]. A summary of the polymers, forms, and drugs targeting various diseases and their delivery is presented in Table 2.

## 6. XG-Based Systems for the Delivery of Genetic Materials

Gene therapy is a novel method that uses genes to treat or prevent various diseases. Instead of employing drugs or surgery to treat a disease, doctors may be able to use the gene therapy procedure to implant a gene into the patient’s cell. Gene therapy involves a variety of techniques, such as (i) “knocking out” or inactivating a defective gene, (ii) gene replacing a disease-causing mutant gene with a healthy gene, and (iii) introducing new genes to guard against any disease [111]. The grafting concept enables three-dimensional interaction with anionic surfaces of double-strand DNA chains by attaching side chain oligomers to either linear or branched hydrophilic polysaccharides. The polysaccharide-based gene delivery technology suggests that the cationic polysaccharide’s structure has a vital impact on the transfection ability for gene delivery. The delivery of complexes such as peptides, oligonucleotides, proteins, and plasmids via the mucosa has been explored using colloidal polysaccharide particles [112]. To ensure a negative charge and hydrophilic nature on the surface of the shell for stabilisation and inherent capability to target endothelial cells, xanthan gum was incorporated in sorbitan monooleate nanoparticles.

**Figure 5:**
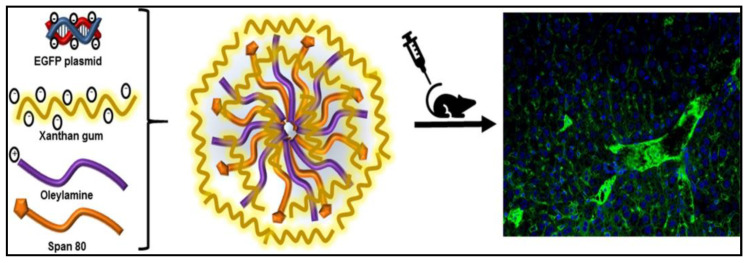
Xanthan gum-based span nanoparticles for gene delivery to endothelial cells [113]. Reproduced with permission from ref [113]. Copyright 2018 Elsevier.

The goal of this work was to add XG into created sorbitan monooleate nanoparticles to target liver sinusoidal endothelial cells (LSECs) by utilising the polysaccharide composition [113]. It was hypothesised that the mannose residues in XG composition would help target the mannose receptors, which are known to be overexpressed in LSECs. As a scavenger receptor, the mannose receptor recognises and promotes the absorption of a variety of glycoconjugate ligands [86]. However, after systemic injection, it was observed that XG span nanoparticles are internalised not only by LSECs but also by vascular endothelial cells that lack the mannose receptor. Enhanced green fluorescent protein plasmid was encapsulated into the nanoparticles. The cytotoxicity and transfection of the loaded nanoparticles were tested against human umbilical vein endothelial cells. Biocompatibility studies were carried out to confirm the biocompatibility of XG-functionalised nanoparticles with vascular endothelium of the liver, lung, and kidney [113]. Figure 5 illustrates the composition of XG-based span nanoparticles for the targeted gene delivery to LSECs.

## 7. XG-Based Systems for the Delivery of Proteins and Peptides

Since they are more effective and have lower toxicity than chemical medications, protein- and peptide-based therapeutics offer a wide range of possibilities as therapeutic agents. However, their use has been constrained due to delivery issues. Their oral bioavailability is very low, and transdermal administration is subject to absorption restrictions. Consequently, parenteral administration is used to deliver the majority of protein- and peptide-based medications. The discomfort of the patient is one issue with this method, particularly for paediatric use. Over the last few decades, intensive research has been conducted to create protein and peptide carriers that overcome the aforementioned issues. Nanoparticle carriers, absorption boosters, enzyme inhibitors, mucoadhesive polymers, and chemical alteration of protein or peptide structures are just a few of the methods that have been used [114]. Short half-life and physical-chemical changes in the protein structures in the gastrointestinal tract cause poor absorption. Crosslinked XG-poly(N-vinyl imidazole) based hydrogel was used to enhance the efficacy of bovine serum albumin (BSA). The carrier used was pH-responsive; further, drug release studies were performed in different pH mediums. The results showed that % release was faster at physiological pH (pH 7.4) than in an acidic medium (pH 1.2) following the non-Fickian release model. The intact structure of the released protein was confirmed by SDS-PAGE, and no disintegration of protein structure was observed. The loaded carrier was non-toxic against normal cells. Protein-loaded graft also showed antimicrobial activity against *Staphylococcus aureus* and *Aspergillus niger* and confirmed their utility as antimicrobial drug carriers [115]. Another carrier system prepared for the delivery of BSA was XG-PVI (poly-N-vinyl imidazole) hydrogel. The drug encapsulation and drug release were directly proportional to the gelling time and concentration of the drug, whereas they were inversely proportional to the concentration of the polymer. While in phosphate-buffered saline (PBS), the release of BSA was increased with the increased concentration of polymer. Cytotoxicity studies showed good biocompatibility of the hydrogel. Furthermore, structure intactness was confirmed by sodium dodecyl-sulfate polyacrylamide gel electrophoresis (SDS-PAGE). Given the results, it was concluded that XG-based drug delivery systems could be used for the delivery of proteins and peptides [116].

## 8. Conclusions and Future Perspective

Xanthan gum is a heteropolysaccharide produced by the fermentation of *X. campestris.* XG being inert and biocompatible in nature is used as an excipient for the sustained and controlled release of drugs. XG is also used as a reducing and capping agent in nanoparticle synthesis. Different forms (hydrogels, matrix tablets, gels, mucoadhesive patches, etc.) of XG are synthesised for the delivery of drugs for the treatment of various diseases. XG has shown applications in different industries, such as food, cosmetics, paints, ceramic glazes, and water-based drilling fluids. It is commercially used as a thickener and stabiliser. Inadequate thermal and mechanical qualities, microbiological contamination, unstable viscosity, low shear resistance, limited surface area, and an unpredictable degree of hydration are a few drawbacks of XG. Additionally, XG disintegrates slowly, particularly in cold water and at high concentrations. It disperses as fish eyeballs, which are water-based lumps, as a result of poor hydration. As a result, the surface of the particles quickly forms a gelatinous surface layer following dispersion. Water cannot pass through the gelatinous barrier, which delays the particle’s complete disintegration. The improvement in physical and chemical characteristics (solubility, swelling, metal-induced gelling ability, mechanical and thermal stability) of XG is achieved by chemical alteration in the number of carboxyl and hydroxyl groups present in its structure. Etherification, esterification, acetylation, oxidation, peptide joining, ionic and covalent crosslinking, and mechanical modification are among the chemical modifications of XG, which might improve its overall physicochemical properties and broaden its applications.

The use of xanthan in the form of such luminous composites has immense promise for biomedical applications. Researchers may be challenged and inspired to develop new structures and functional systems based on xanthan gum because it is a fascinating biopolymer with a variety of intriguing features.

## Figures and Tables

**Table 1:** Physicochemical properties of Xanthan gum.

Properties	Value
Physical state	Dry-solid powder
Colour	Yellow-white
Molecular weight	Ranges 1 × 10^6^ to 2 × 10^6^ g/mol
pH	6–7
Flash point	>100 °C
Solubility	Water, DMSO, DMF

**Table 2:** Summary of polymers, dosage forms, drugs, and their delivery.

Sr. No.	Polymers	Form	Drug	Remarks	Reference
***Delivery of antimicrobial drugs***					
1.	XG, sodium alginate and ethyl cellulose	In situ ocular gels	Besifloxacin	The transparent, clear, non-irritant gel was synthesised with sustained drug release (for 8 h) and high drug content capacity.	[44]
2.	XG, HPMC, and sodium alginate	In situ ocular gels	Flucytosine	The drug-loaded formulation showed high drug content (95.2–98.6%), sustained drug release (up to 8 h) and stability at both room and higher temperatures.	[45]
3.	XG, HPMC, and sodium alginate	Floating low-density tablet	Stavudine	Sustained release of the antiviral drug stavudine was achieved with all the pre-compression and post-compression characteristics for the tablet.	[46]
4.	Xanthan gum, bovine serum albumin and magnetic nanoparticles	Magnetic field responsive antimicrobial patch	Amoxicillin	The adsorption of the drug was found to be more on XG and BSA films compared to others. Drug release followed a quasi-Fickian pattern. The formulation was tested for antimicrobial activity, and it showed more efficacy in the presence of an external magnetic field.	[100]
5.	XG		Combination of tobramycin and dexamethasone	The combination of both drugs loaded into xanthan gum was better than the commercially available treatment even when it contains half the amount of steroid.	[101]
6.	XG coated with Eudragit-L 100	Tablets	Metronidazole	Coated XG tablets showed delayed release. In the dissolution medium, decreased drug release was observed due to microbial degradation or polymer solubilisation. The release of the drug was greatly affected by the nature of the polymer used and the enteric coating of the tablet.	[102]
7.	XG and Chitosan	Polyelectrolyte hydrogel	Ciprofloxacin	Increased drug encapsulation with an increase in drug concentration was observed, and it followed zero-order drug release kinetics. The drug-loaded hydrogel inhibited the growth of bacteria and showed no effect against fungi.	[103]
8.	XG	Microspheres	Ciprofloxacin	The in-vitro drug release was carried out in both acidic as well as alkaline mediums; the release pattern followed a non-Fickian trend. Based on the results, it was concluded that the synthesised formulation was suitable for the sustained release of ciprofloxacin.	[104]
9.	XG	Hydrogel	Liranaftate	The microemulsion-based drug hydrogel showed increased antifungal activity in comparison to plain drug solution against *Candida albicans*. The microemulsion proved to increase the skin retention and permeability of the drug.	[105]
10.	XG, HPMC K15, and HPMC K100M	Floating low-density tablet	Stavudine	An increase in the half-life of stavudine in the gastric fluid was noted. Sustained release of the antiviral drug stavudine was achieved with all the pre-compression and post-compression characteristics.	[46]
11.	XG with magnetic nanoparticles (Fe_3_O_4)_	Hydrogel	Terbinafine	The hydrogel shows thermally induced controlled drug delivery (3-fold) by magnetic hyperthermia and high efficacy compared to the pure drug for antifungal properties. The incorporation of Fe_3_O_4_ nanoparticles helps the hydrogel function as non-invasive monitoring by magnetic resonance imaging (MRI).	[47]
12.	XG, poly(-vinyl alcohol), sodium alginate	Interpenetrating polymer network microbeads	Norfloxacin	Hydrogel showed high encapsulation efficiency. The microbeads had mucoadhesive properties at physiological pH, which was assessed with the use of a caprine intestine. The system showed pH-dependent drug release; it fitted the Higuchi and Korsemeyer–Peppas models.	[106]
13.	XG and Chitosan	Hydrogel	Acyclovir	The drug-loaded formulation showed pH-dependent drug release and swelling behaviour; it fitted the Higuchi and Korsemeyer–Peppas models. It was observed that the hydrogel had a porous structure and was thermally stable.	[49]
14.	XG and Chitosan	Hydrogel scaffold	Ampicillin, Rifampicin, and Minocycline.	Hydrogel showed good swelling and porosity. The in vitro drug release study of all the antibiotics showed fast drug release within 24 h in simulated gastric fluid. The gels prepared were stated to be non-toxic after the in vitro cell cytocompatibility test. Antibacterial activity of the loaded formulation was evaluated.	[107]
15.	XG	Solution	Linezolid	The drug-loaded formulation was analysed against *Staphylococcus aureus* infections in the ocular tissues. The in vitro antibacterial activity and in vivo evaluation of drug efficacy showed that the linezolid-loaded XG exhibits better topical instillation and ocular permeation with bioadhesion to the precorneal membrane.	[108]
***Delivery of chemotherapeutic drugs***					
1.	XG	Microspheres and Hydrogels	α-linolenic acid and Docosahexaenoic acid	The antioxidant activity and anti-neoplastic activity of α-linolenic acid were increased when loaded into the XG-based nanoformulations whereas the activity of docosahexaenoic acid remained the same when treated against colon cancer.	[50]
2.	XG-chitosan	Nanofibers	Curcumin	The developed nanofibers were observed to be stable at pH 6.5 and pH 7.4. The drug-loaded formulation was tested on the colon cancer cell line; it was observed that the loaded polyphenol showed more efficacy than the pure drug. The permeation of the drug was increased by 3.4-fold.	[53]
3.	XG	pH-responsive nanoparticles	Doxorubicin	The nanoparticles showed high drug loading and pH-sensitive drug release. The drug-loaded formulation was biocompatible.	[54]
4.	XG	Nanogels	Doxorubicin	Nanogels were also nontoxic and biocompatible proving to be an ideal carrier for the delivery of chemotherapeutic drugs.	[55]
5.	XG coated Gold nanoparticles	-	Doxorubicin	XG is used as a stabilising and reducing agent. In vitro studies showed that nanoparticles coated with XG showed a 3-fold increase in the efficacy of the drug against the A549 cell line.	[56]
6.	Carboxymethyl XG coated Gold nanoparticles	-	-	Modified XG was used as a stabilising agent. The release of the drug was extensive at acidic pH and physiological pH. In-vitro studies of drug-loaded nanoparticles showed a 4.6-fold increase in the anticancer activity of the drug compared to pure drug.	[57]
7.	XG-poly(-vinylpyrrolidone-co-poly)-acrylic acid	pH-responsive hydrogel	5-fluorouracil	Controlled release of the drug was observed. It also showed stability in a wide range of pH media and maximum swelling in basic medium confirming its use as a pH-responsive hydrogel. In vivo studies showed acute oral toxicity studies were performed.	[52]
8.	XG and guar gum	Nanoparticles	5-fluorouracil	In vitro and in vivo studies showed intact microflora of the intestine, which fulfils the aim of the research of overcoming the side effects of 5-fluorouracil by co-administration of probiotics (*B. bifidum*)	[51]
9.	XG	Thermoreversible gel	Ondansetron hydrochloride	The addition of XG increased the viscosity of the gel, thus decreasing the release of the drug and achieving sustained drug release. The gel was stable at room temperature and freeze temperature for a period of a month.	[109]
***Delivery of anti-diabetic drugs***					
1.	XG	Topical nanoemulgel	Glimepiride	XG (3% *w/w*) was used as a gelling agent. The formulation significantly increased the permeation and bioavailability of the drug.	[62]
2.	XG	Mouth dissolving films	Glibenclamide	The films were formed for the rapid release of the drug and to increase its bioavailability in the body. Stability studies proved the formulation was stable even in extreme conditions.	[37]
3.	XG and HPMC K100M	Mucoadhesive buccal tablet	Repaglinide	The drug release was directly proportional to the concentration of both the polymers used. The stability studies showed that the tablets were stable for up to 2 months without any decrease in the drug content.	[63]
4.	XG stabilised gold nanoparticles		Tolbutamide	The loaded formulation showed high loading efficiency without changing the structure of the drug. Drug-loaded formulation was investigated for its insulin secretion in mice models; it enhanced insulin secretion when compared to the plain drug solution.	[64]
***Delivery of drugs for the treatment of cardiovascular disease***					
1.	XG, HPMC, and corn starch	Matrix	Verapamil hydrochloride	XG-based formulation significantly provided sustained release of the drug. The drug release was dependent upon the concentration of the polymer.	[65]
2.	XG and Chitosan	Tablets	Propranolol hydrochloride	The mixture of both polymers prolonged the time of drug release from the matrix at pH 1.2. Three layered polymer tablet pH-responsive was formed, with an increase in the barrier/middle layer of the matrix, and the lag in the release also increased.	[66]
3.	XG, HPMC K4M, and sodium alginate	Transdermal patches	Carvedilol	The XG-based formulation showed drug release within 12 h. It was stable in various physicochemical parameters and ideal for the delivery of carvedilol.	[67]
4.	XG and poly(-vinyl alcohol)	Membranes	Diltiazem HCl	Mechanical strength, physical properties, and drug release were observed to be dependent on the molecular weight of the polymers used for the preparation of membranes.	[68]
***Anti-spasmodic drug delivery***					
1.	XG and sodium alginate	Floating tablets	Fenoverine	The formulation with 15% XG was ideal, with 99.6% drug release in 12 h. It also showed an improved swelling ratio with required drug release kinetics and floating behaviour.	[72]
2.	XG	Matrix Tablet	Mebeverine HCl	XG can hydrate more rapidly than the other three gums used. The resulting drug diffusional path length for XG was the longest. The in vitro drug release studies revealed that the level of the polymer in the matrix tablets played an important role in the modulation of drug release.	[110]
***Delivery of drugs for the treatment of inflammation, rheumatoid arthritis, and gout***					
1.	XG and graphene oxide	Hydrogel	Diclofenac potassium	The swelling ratio and drug release certainly increased with the increase in pH. The drug-loaded composite hydrogel was pH-responsive. In vivo studies concluded the bioavailability of the drug increased with an increase in the concentration of the composite hydrogel.	[75]
2.	Carboxylated XG	Hydrogels	Diclofenac	An increase in viscoelasticity was observed due to the presence of XG in the system. The encapsulation efficiency of the drug was increased and the sustained release of the drug was due to the presence of nanotubes. The composite was stable in extreme conditions similar to that may be present on the skin.	[76]
3.	Thiolated XG	Topical gel	Lornoxicam	The physicochemical properties of the gel formed were ideal. The high drug content in the thiolated gel was observed. In-vitro drug release studies showed sustained release up to 24 h. The thiolated gel was ideal for topical drug delivery for the treatment of inflammation and pain.	[33]
4.	XG and *Vigna mungo*	Mucoadhesive bilayer buccal tablets	Febuxostat	The tablets were used to increase the bioavailability of febuxostat at the mucosal site for a prolonged time. The sustained release of the drug occurred for 8 h. The tablets were stable at room temperature and showed good mucoadhesive properties.	[77]
5.	XG and chitosan	Microparticles	Quercetin	The microparticles were loaded with quercetin and the release was controlled by non-Fickian diffusion of the drug. The microparticles were compressed and converted into tablets with a coating of Eudragit.	[78]
6.	XG and ethyl cellulose	Matrix tablet	Metoclopramide	Pre-compression and post-compression characteristics of the tablets were found to be ideal. The tablets prepared using XG and XG-ethyl cellulose both showed sustained drug release for up to 12 h.	[79]
7.	Polyacrylamide-grafted-XG	pH-responsive nanoparticles	Curcumin	In an alkaline medium, the drug release was excellent, with the presence of rat caecal suggesting microflora-dependent drug release. In vivo studies suggested that the drug-loaded nanoparticles showed better anti-inflammatory activity when compared to the pure drug.	[80]
***Delivery of immunosuppressive drugs***					
1.	XG, Carbopol and gellan gum	In-situ ocular gel	Ketotifen fumarate	pH-sensitive ocular gels were prepared for better efficacy than the formulation present in the market. Different concentrations of polymers: xanthan gum, Carbopol^®^, and gellan gum were taken to form different formulations. Among the optimised formulations, formulation with 0.75% Carbopol and 0.3% xanthan gum was found to be the best. It had a drug encapsulation efficiency of 99.74%, gel-forming strength in 46.6 s, and drug release of up to 8 h.	[83]
2.	XG and sodium alginate	Microbeads	Tacrolimus	The sodium alginate was taken as the main polymer but to increase the drug-loading efficiency of microbeads, xanthan gum was added to the formulation. Using different concentrations of crosslinkers also affects drug release. Also, the drug release is dependent on the concentration of the polymer and coating polymer. It was suggested that this system could be used as an alternative and cost-effective system for the delivery of drugs.	[85]
***Delivery of drugs for the treatment of skin diseases***					
	Sodium alginate and XG	Bioadhesive film	Pycnogenol	The film showed suitable mechanical properties such as high deformability, suggesting easy adaptability to any type of surface. The film shows high bioadhesion on the skin and absorption of exudates from the open wound. The drug-loaded films can inhibit the growth of bacterial strains.	[87]
***Delivery of drugs for the treatment of central nervous system-related disorders***					
1.	XG, Carbopol 934P, HPMC K4M, and polyvinyl pyrrolidone K30	Mucoadhesive matrix tablet	Quetiapine fumarate	Sustained release (96%) was obtained for a prolonged time (10 h). The permeation in the mucus membrane was 77%.	[89]
2.	XG and tragacanth	Tablets		XG exhibited a swelling mechanism for the controlled release of the drug. The binary tablets formed with XG showed pH-responsive drug release, fitting Higuchi and Korsmeyer–Peppas models.	[90]
3.	XG, guar gum, karaya gum	Oral tablets	Zolmitriptan	Formulations containing xanthan gum showed an increase in sustained release of the drug with the increase in the concentration of polymer.	[91]
4.	xanthan gum and gellan gum	In-situ gel	Resveratrol	The optimisation of in situ gel resulted in 5-fold higher permeation in the nasal mucosa when compared to pure drug suspension-based gel. The drug-loaded formulation was evaluated using the scopolamine-induced amnesia model in mice. Improvement in memory function was observed.	[92]
***Delivery of drugs for the treatment of obesity***					
	Xanthan gum	Mini-tablets	Orlistat	The mini-tablets formed using XG as an oil-reducing agent might help in the development of many other delivery systems for anti-obesity drugs for reducing their side effects.	[93]
***Delivery of drugs for the treatment of glaucoma***					
1.	Pure gellan gum and in combination with HPMC, XG, and Carbopol	pH-responsive in situ gel	Acetazolamide	A combination of XG with gellan gum proved to be the best formulation among all, increasing mucoadhesive properties and a relative decrease in ocular pressure when compared to commercial oral tablets and eye drops.	[94]
2.	XG and poly acrylic acid	Mucoadhesive in situ gel	Dorzolamide hydrochloride	Formulation with 0.2% *w/v* XG showed 90.84% of drug release for 12 h. The drug release was also analysed using goat’s cornea; formulation showed similar release kinetics to the in vitro release for up to 9 h.	[96]
***Delivery of drugs for the treatment of pulmonary diseases***					
1.	Sodium carboxymethyl XG and casein crosslinked with aluminium chloride and glutaraldehyde	Interpenetrating polymer network-based hydrogel beads	Theophylline	Dissolution studies and drug release carried out in both acidic and alkaline mediums delayed release of the drug was observed.	[98]
2.	XG	Mini-tablets and matrix tablets.	Theophylline	It increased the solubility of the drug by 2.8 times in 40% alcohol. At lower concentrations of XG with large particles, the drug release was observed to be faster when compared to the higher concentration of polymer with finer particles (<75 µm) in dissolution studies.	[99]

## Data Availability

As no new data were created in this article, data sharing is not applicable to this article.
